# Association of breast cancer with quantitative mammographic density measures for women receiving contrast-enhanced mammography

**DOI:** 10.1093/jncics/pkae026

**Published:** 2024-04-02

**Authors:** Gordon P Watt, Krishna N Keshavamurthy, Tuong L Nguyen, Marc B I Lobbes, Maxine S Jochelson, Janice S Sung, Chaya S Moskowitz, Prusha Patel, Xiaolin Liang, Meghan Woods, John L Hopper, Malcolm C Pike, Jonine L Bernstein

**Affiliations:** Department of Epidemiology and Biostatistics, Memorial Sloan Kettering Cancer Center, New York, NY, USA; Department of Radiology, Memorial Sloan Kettering Cancer Center, New York, NY, USA; Melbourne School of Population and Global Health, University of Melbourne, Parkville, VIC, Australia; Department of Medical Imaging, Zuyderland Medical Center, Sittard-Geleen, The Netherlands; Department of Radiology, Memorial Sloan Kettering Cancer Center, New York, NY, USA; Department of Radiology, Memorial Sloan Kettering Cancer Center, New York, NY, USA; Department of Epidemiology and Biostatistics, Memorial Sloan Kettering Cancer Center, New York, NY, USA; Department of Epidemiology and Biostatistics, Memorial Sloan Kettering Cancer Center, New York, NY, USA; Department of Epidemiology and Biostatistics, Memorial Sloan Kettering Cancer Center, New York, NY, USA; Department of Epidemiology and Biostatistics, Memorial Sloan Kettering Cancer Center, New York, NY, USA; Melbourne School of Population and Global Health, University of Melbourne, Parkville, VIC, Australia; Department of Epidemiology and Biostatistics, Memorial Sloan Kettering Cancer Center, New York, NY, USA; Department of Epidemiology and Biostatistics, Memorial Sloan Kettering Cancer Center, New York, NY, USA

## Abstract

Women with high mammographic density have an increased risk of breast cancer. They may be offered contrast-enhanced mammography to improve breast cancer screening performance. Using a cohort of women receiving contrast-enhanced mammography, we evaluated whether conventional and modified mammographic density measures were associated with breast cancer. Sixty-six patients with newly diagnosed unilateral breast cancer were frequency matched on the basis of age to 133 cancer-free control individuals. On low-energy craniocaudal contrast-enhanced mammograms (equivalent to standard mammograms), we measured quantitative mammographic density using CUMULUS software at the conventional intensity threshold (“Cumulus”) and higher-than-conventional thresholds (“Altocumulus,” “Cirrocumulus”). The measures were standardized to enable estimation of odds ratio per adjusted standard deviation (OPERA). In multivariable logistic regression of case-control status, only the highest-intensity measure (Cirrocumulus) was statistically significantly associated with breast cancer (OPERA = 1.40, 95% confidence interval = 1.04 to 1.89). Conventional Cumulus did not contribute to model fit. For women receiving contrast-enhanced mammography, Cirrocumulus mammographic density may better predict breast cancer than conventional quantitative mammographic density.

High mammographic density is associated with future breast cancer risk (1,2). Mammographic density can be assessed qualitatively by radiologists (3) or quantitatively (4-6). CUMULUS software (4) is a widely used quantitative method by to measure mammographic density wherein the user separates the radiodense (bright) from radiolucent (dark) areas of the mammogram, outputting absolute mammographic dense area and mammographic percentage density. Cumulus mammographic dense area and mammographic percentage density are associated with breast cancer in diverse populations (2,4,5-13). Additionally, modified CUMULUS-based measures of mammographic density using higher-than-conventional intensity thresholds may improve the discrimination of breast cancer risk relative to conventional Cumulus mammographic density (8-13).

For the 48% of the US population with “heterogeneously”/“extremely” dense breasts (14), mammography has lower sensitivity to detect breast cancer than in women with “almost entirely fatty” breasts or “scattered areas of fibroglandular density” (15). For these women, and for those women with intermediate lifetime risk (15%-19%) of breast cancer, dual-energy contrast-enhanced mammography may be incorporated to improve screening performance (16-19). After administration of an iodine-based contrast agent, 2 images at low and high energy levels are captured. The low-energy image is equivalent to a standard full-field digital mammogram (20) and enables measurement of mammographic density. The high-energy and low-energy images are processed to produce a recombined image that improves screening performance (17,19,21). In the population receiving contrast-enhanced mammograms, it is not clear how conventional and modified measures of mammographic density contribute to breast cancer risk assessment.

We evaluated whether quantitative mammographic density measures from low-energy contrast-enhanced mammograms were associated with risk of newly diagnosed invasive breast cancer in a case-control study of women receiving contrast-enhanced mammograms. Eligible women received a bilateral contrast-enhanced mammogram at Memorial Sloan Kettering Cancer Center from 2010 to 2020. Women with a history of breast cancer were excluded. For women with more than 1 contrast-enhanced mammogram, the earliest image was taken. From 959 eligible women, 68 (7.2%) were newly diagnosed with invasive unilateral breast cancer at the time of or after the contrast-enhanced mammogram (cases). The 68 women were frequency matched by age to 136 cancer-free women (controls) with no diagnosis of breast cancer through 2021. Controls were selected randomly within 5-year age groups to match the distribution of cases. Medical record data of the selected case-control patients were abstracted into a Research Electronic Data Capture database (22). During medical record review, we excluded 2 patients with ductal carcinoma in situ without invasive components, in accordance with the study protocol. Three controls with unusable images were excluded, leaving a final sample of 66 cases and 133 controls (Supplementary Figure 1, available online). The study received ethical approval from the Institutional Review Board at Memorial Sloan Kettering Cancer Center with a waiver of informed consent.

For cases, we analyzed the unaffected contralateral breast; for controls, we analyzed a randomly selected laterality. Experienced users of CUMULUS software assessed deidentified craniocaudal view low-energy images, with blinding to patient characteristics. High-energy and recombined images were not used. We assessed mammographic dense area at the conventional intensity threshold (Cumulus) and mammographic dense area at 2 higher-intensity thresholds: Altocumulus, capturing the bright as opposed to white areas, and Cirrocumulus, capturing only the brightest areas within the bright areas (8,12). We calculated mammographic percentage density as mammographic dense area divided by total breast area and breast fat area as total breast area minus Cumulus mammographic dense area.

Using the images from the 755 cancer-free women not selected for the case-control study, we trained an nnU-net model (23,24) to segment mammographic dense area. The model was trained and tuned on Cumulus mammographic dense areas measured on the 755 cancer-free images, and the accuracy was tested on Cumulus mammographic dense areas measured on the 199 images from the case-control sample (holdout images). In the 199 holdout images, we assessed pixel-by-pixel overlap using the Dice Score Coefficient and consistency using the 2-way intraclass correlation coefficient (25); we checked for systematic trends using a Bland-Altman plot. The nnU-net was developed in Python using the PyTorch, NumPy, SciPy, and nnUNet packages.

Age and adiposity are known confounders of the association between breast density and breast cancer (26). Each mammographic density measure was thus parameterized to enable estimation of the odds ratio per adjusted standard deviation (OPERA) (27). The adjusted residuals were calculated for the controls by regressing each measure on age and breast fat area. The mammographic density measures were then standardized per standard deviation of the adjusted residuals. Breast fat area was used because body mass index was not universally available in the medical record. For the 182 individuals with body mass index data, the correlation between breast fat area and body mass index was 0.79.

We estimated the associations of breast cancer case-control status with each mammographic percentage density measure (Cumulus, Altocumulus, Cirrocumulus, and nnU-net) using multivariable logistic regression. Models were fit for each mammographic percentage density measure separately, then in combination, as in prior work (8,9,12). In the models fitting multiple CUMULUS-based measures together, the measures were modified to represent mutually exclusive ranges of intensities (12). Models were adjusted for age at time of contrast-enhanced mammogram, year of contrast-enhanced mammogram, square root of breast fat area, *BRCA1*/*BRCA2* variations (negative, positive, or not tested), history of lobular carcinoma in situ, age at menarche (<13 vs ≥13 years), menopausal status at the time of contrast-enhanced mammogram (premenopausal, postmenopausal), and first-degree family history of breast cancer. The missing covariate indicator method was used for observations missing covariate values (28). Case-control discrimination for each measure was determined by comparing the OPERA estimates. Model fits were compared using Akaike information criterion (AIC). In sensitivity analyses, we 1) repeated the analysis restricted to cases diagnosed more than 60 days after the contrast-enhanced mammogram, effectively excluding contrast-enhanced mammograms acquired during diagnostic workup, and 2) refit the multivariable models using mammographic dense area measures rather than mammographic percentage density. Statistical analyses were completed using R (R Foundation for Statistical Computing, Vienna, Austria). The 2-way type I error rate for statistical tests and confidence intervals (CIs) was 0.05.

Supplementary Table 1 (available online) shows that participants were well matched on age at time of contrast-enhanced mammogram and had a similar distribution by menopausal status. Forty-three (65%) of cases were diagnosed within 2 months after the contrast-enhanced mammogram, and 23 (35%) were diagnosed more than 60 days after the contrast-enhanced mammogram. The proportion with a family history of breast cancer or personal history of lobular carcinoma in situ or benign breast disease was higher than the general population, reflecting the population eligible for contrast-enhanced mammogram.

The nnU-net measure had a Dice Score Coefficient of 0.94 (range = 0.66-0.99) compared with the Cumulus ground truth. The intraclass correlation coefficient was 0.94 (95% CI = 0.92 to 0.95). A Bland-Altman plot comparing the square root–transformed Cumulus mammographic dense area and nnU-net mammographic dense area is given in Supplementary Figure 2 (available online).

Figure 1, A and B show that nnU-net mammographic percentage density had a strong correlation with conventional Cumulus mammographic percentage density (Pearson *r* = 0.87) and a weak correlation with Cirrocumulus mammographic percentage density (*r* = 0.23), respectively.

**Figure 1.**
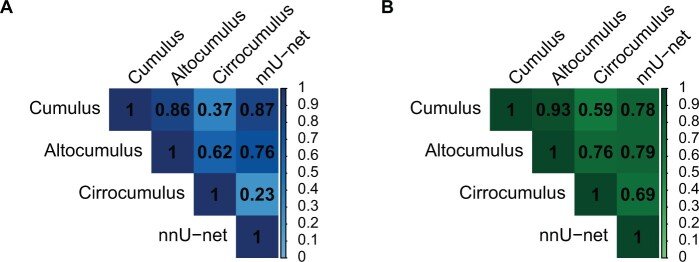
Pearson correlations between the (**A**) mammographic percent density and (**B**) mammographic dense area risk measures derived from low-energy contrast-enhanced mammograms. Cumulus, Altocumulus, and Cirrocumulus are measured using the semiautomated thresholding software CUMULUS. “Cumulus” refers to the conventional definition of breast density, capturing all the white or bright areas on the image; “Altocumulus” captures only the bright areas; and “Cirrocumulus” captures only the brightest areas. Examples are provided in prior publications (8,12). nnU-net is a fully automated measure trained on CUMULUS estimates in a separate set of low-energy contrast-enhanced mammograms. Each measure was standardized per odds ratio per adjusted standard deviation for the controls.

Table 1 displays the results from the case-control regressions. In models fitting each mammographic percentage density measure separately, case-control status was associated with Cirrocumulus mammographic percentage density (odds ratio [OR] = 1.40, 95% CI = 1.04 to 1.89). The Cirrocumulus-only model had the best model fit (AIC = 249.3), with the AIC 5 points lower than the model fitting conventional Cumulus mammographic percentage density alone (OR = 0.99, 95% CI = 0.70 to 1.40; AIC = 254.3). Models fitting Altocumulus mammographic percentage density with the other measures resulted in high multicollinearity (variance inflation factor >5) and were not considered further. When fitting Cirrocumulus mammographic percentage density together with Cumulus mammographic percentage density, the association between case-control status and Cirrocumulus mammographic percentage density was unchanged, and model fit was not improved (AIC = 250.4). The association between case-control status and the nnU-net mammographic percentage density measure was likewise not statistically significant and did not improve model fit when fit together with Cirrocumulus mammographic percentage density. In our sensitivity analysis restricted to the 23 cases diagnosed more than 60 days after the contrast-enhanced mammogram, the association between case-control status and Cirrocumulus mammographic percentage density was similar but statistically nonsignificant (OR = 1.57, 95% CI = 0.92 to 2.69).

**Table 1.** Multivariable models of the association between breast cancer and mammographic percentage density measures from low-energy contrast-enhanced mammograms

**Measure** ^a^	**Odds ratio per adjusted standard deviation** ^b^	95% Confidence interval	‒2 Log likelihood	Akaike information criterion
**Each mammographic percentage density measure in separate models**				
Cirrocumulus	1.40	1.04 to 1.89	215.3	249.3
Altocumulus	1.19	0.87 to 1.64	219.2	253.2
Cumulus	0.99	0.70 to 1.40	220.3	254.3
nnU-net	1.01	0.71 to 1.44	220.3	254.3
**Cirrocumulus and Cumulus mammographic percentage density fit in same model^c^**				
Cirrocumulus	1.43	1.06 to 1.94	214.4	250.4
Cumulus	0.84	0.59 to 1.21	—	—
**Cirrocumulus and nnU-net mammographic percentage density fit in same model^d^**				
Cirrocumulus	1.38	1.01 to 1.87	216.6	252.6
nnU-net	0.89	0.60 to 1.30	—	—

a Measures were log-transformed and standardized by age-adjusted and the square root of the breast fat area minus–adjusted SD for the controls (27).

b Models were adjusted for age at time of contrast-enhanced mammogram; year of contrast-enhanced mammogram; race and ethnicity; age at menarche (<13 vs ≥13 years); square root of breast fat area in centimeters squared; history of testing for and presence of pathogenic alterations in *BRCA1* or *BRCA2*; and history of atypical ductal hyperplasia, atypical lobular hyperplasia, and lobular carcinoma in situ. The missing covariate indicator method was used to fill covariates with missing values.

c CUMULUS-based measures were re-paramaterized as mutually exclusive ranges of intensities before fitting together in multivariable model.

d nnU-net is a fully automated measure of mammographic density trained on conventional Cumulus as the ground truth on a separate set of 755 low-energy contrast-enhanced mammogram images.

Supplementary Table 2 (available online) shows model fits using mammographic dense area rather than mammographic percentage density. Cirrocumulus mammographic dense area had a positive association with breast cancer (OR = 1.33, 95% CI = 1.00 to 1.78); the other measures did not improve model fit (ΔAIC < 1).

This study suggests that the highest-intensity measure of mammographic density, Cirrocumulus, is superior to conventional mammographic density for breast cancer risk assessment for patients receiving contrast-enhanced mammograms. When Cirrocumulus was modelled together with conventional mammographic density measures (Cumulus or nnU-net), the conventional measures did not improve model fit, suggesting that Cirrocumulus captures most of the variation in breast cancer risk attributed to mammographic density. These results reflect results from prior work in populations receiving standard mammogram (8-12). The present study advances the evidence for Cirrocumulus as the preferred mammographic density measure for a sample with higher baseline risk. Further, because low-energy contrast-enhanced mammograms are identical to full-field digital mammograms, our results are generalizable to similar populations receiving full-field digital mammograms instead of contrast-enhanced mammograms.

This study’s strengths include detailed adjustment for clinical and epidemiological factors, the comparison of semiautomated measures, and a fully automated measure of mammographic density. There were several limitations as well. First, the sample included only 66 newly diagnosed invasive cancers from a single institution. Although the single-institution design reduced the heterogeneity in image acquisition, it precluded subgroup analyses. Second, the sample comprised 86% non-Hispanic White patients, which may reduce generalizability to other populations. Third, we included cases diagnosed at the time of and after the contrast-enhanced mammogram date, introducing heterogeneity in the case population. Results were consistent when restricting to cases diagnosed more than 2 months after the contrast-enhanced mammogram, but a longitudinal study of women with cancer-free baseline contrast-enhanced mammograms is warranted.

In conclusion, Cirrocumulus, capturing only the brightest areas of a mammogram, may be a more precise imaging measure for breast cancer risk assessment in the population receiving contrast-enhanced mammograms.

## Supplementary Material

pkae026_Supplementary_Data

## Data Availability

The data that support the findings of this study are available from the corresponding author upon reasonable request. The data are not publicly available due to privacy or ethical restrictions.
