# Supplementary material for: Association of breast cancer with quantitative mammographic density measures for women receiving contrast-enhanced mammography
Source: JNCI Cancer Spectr. 2024 Apr 2;8(3):pkae026. doi: 10.1093/jncics/pkae026 (PMC11060476; doi:10.1093/jncics/pkae026)
Supplement: pkae026_Supplementary_Data [file pkae026_supplementary_data.pdf]

Supplementary Material

Contents

Supplementary Table 1 2
Supplementary Table 2 4
Supplementary Figure 1 5
Supplementary Figure 2 6

Supplementary Table 1

Characteristics of the nested case-control study population, selected from a cohort of patients receiving CEM

Characteristic	Control, N = 133[a]	Case, N = 66
**Age at time of CEM**		
<45	45 (34%)	21 (32%)
46 to <60	63 (47%)	32 (48%)
60+	25 (19%)	13 (20%)
**Menopausal status at time of CEM**		
Postmenopausal	69 (52%)	35 (53%)
Premenopausal	64 (48%)	31 (47%)
**Age at menarche**		
13+	70 (55%)	38 (59%)
<13	57 (45%)	26 (41%)
Unknown	6	2
**Parity**		
Nulliparous	43 (33%)	19 (29%)
1 full-term birth	25 (19%)	11 (17%)
2+ full-term births	64 (48%)	36 (55%)
Unknown	1	0
**Self-reported race**		
Asian	3 (2.3%)	3 (4.5%)
Black	6 (4.5%)	8 (12%)
Other[b]	4 (3.0%)	3 (4.5%)
White	119 (90%)	52 (79%)
Unknown	1	0
**Self-reported ethnicity**		
Not Hispanic	124 (95%)	58 (89%)
Hispanic	7 (5.3%)	7 (11%)
Unknown	2	1
**Year of CEM**		
2010-2013	4 (3.0%)	12 (18%)
2014-2016	82 (62%)	27 (41%)
2017-2020	47 (35%)	27 (41%)
**Family history of breast cancer**		
No family history	37 (28%)	28 (43%)
Any family history	96 (72%)	37 (57%)
Unknown	0	1
**History of ADH**		
No	95 (71%)	55 (83%)
Yes	38 (29%)	11 (17%)
**History of ALH**		
No	116 (87%)	56 (85%)
Yes	17 (13%)	10 (15%)
**History of LCIS**		
No	96 (72%)	49 (74%)
Yes	37 (28%)	17 (26%)
**Any history of invasive cancer**		
No	126 (95%)	61 (92%)
Yes	7 (5.3%)	5 (7.6%)
***BRCA1* mutation**		
Negative	17 (13%)	22 (33%)
Positive	3 (2.3%)	0 (0%)

Characteristic	Control, N = 133[a]	Case, N = 66
Not tested	113 (85%)	44 (67%)
***BRCA2* mutation**		
Negative	14 (11%)	18 (27%)
Positive	6 (4.5%)	4 (6.1%)
Not tested	113 (85%)	44 (67%)

Abbreviations. CEM, contrast-enhanced mammogram; ADH, atypical ductal hyperplasia; ALH, atypical lobular hyperplasia; LCIS, lobular carcinoma in situ

[a] The case-control study population was selected from a cohort of 959 women who received CEM at Memorial Sloan Kettering Cancer Center from 2010-2020 and who had no prior history of breast cancer. Case women received a CEM and a new diagnosis of invasive breast cancer at the time of or after their CEM; control women received a CEM and had no diagnosis of invasive breast cancer in the medical record. Controls were frequency-matched to cases at a 2:1 ratio on age at time of the CEM (5-year matching groups).

[b] More than one race or other self-reported category

Supplementary Table 2

Multivariable models of the association between breast cancer and mammographic dense area (MDA) measured on low-energy contrast-enhanced mammograms

Measures[a]	OPERA[b]	95% Lower	95% Upper	-2(LL)	AIC
Each MDA measure in separate models					
Cirrocumulus	1.33	1.00	1.78	216.6	250.6
Altocumulus	1.16	0.85	1.57	219.5	253.5
Cumulus	1.00	0.72	1.41	220.3	254.3
nnU-net	1.23	0.87	1.74	218.9	253.0
Cirrocumulus and Cumulus MDA fit in same model[c]					
Cirrocumulus	1.54	1.10	2.18	213.9	249.9
Cumulus	0.71	0.47	1.08		
Cirrocumulus and nnU-net MDA fit in same model[d]					
Cirrocumulus	1.37	0.92	2.05	216.6	252.6
nnU-net	0.95	0.59	1.54		

Abbreviations. OPERA, odds per adjusted standard deviation; LL, log-likelihood; AIC, Akaike's Information Criterion; MDA, mammographic dense area

[a] Measures were log-transformed and standardized per age- and sqrt(breast fat area)-adjusted standard deviation for the controls (Hopper *et al. Amer J Epidemiol* 2015)

[b] Odds per age- and sqrt(breast fat area)-adjusted standard deviation. Models were adjusted for age at time of CEM; year of CEM exam; race/ethnicity; age at menarche (≥ 13 vs < 13 years); square root of breast fat volume (cm^2); history of testing for and presence of pathogenic mutations in *BRCA1* or *BRCA2*; and history of atypical ductal hyperplasia, atypical lobular hyperplasia, and lobular carcinoma *in situ*. The missing covariate indicator method was used to fill covariates with missing values

[c] CUMULUS-based measures were re-paramaterized as mutually-exclusive areas of density prior to fitting together in multivariable model

[d] nnU-net is a fully automated measure of mammographic density trained on conventionaal Cumulus as the ground truth on a separate set of 755 low-energy CEM images

Supplementary Figure 1

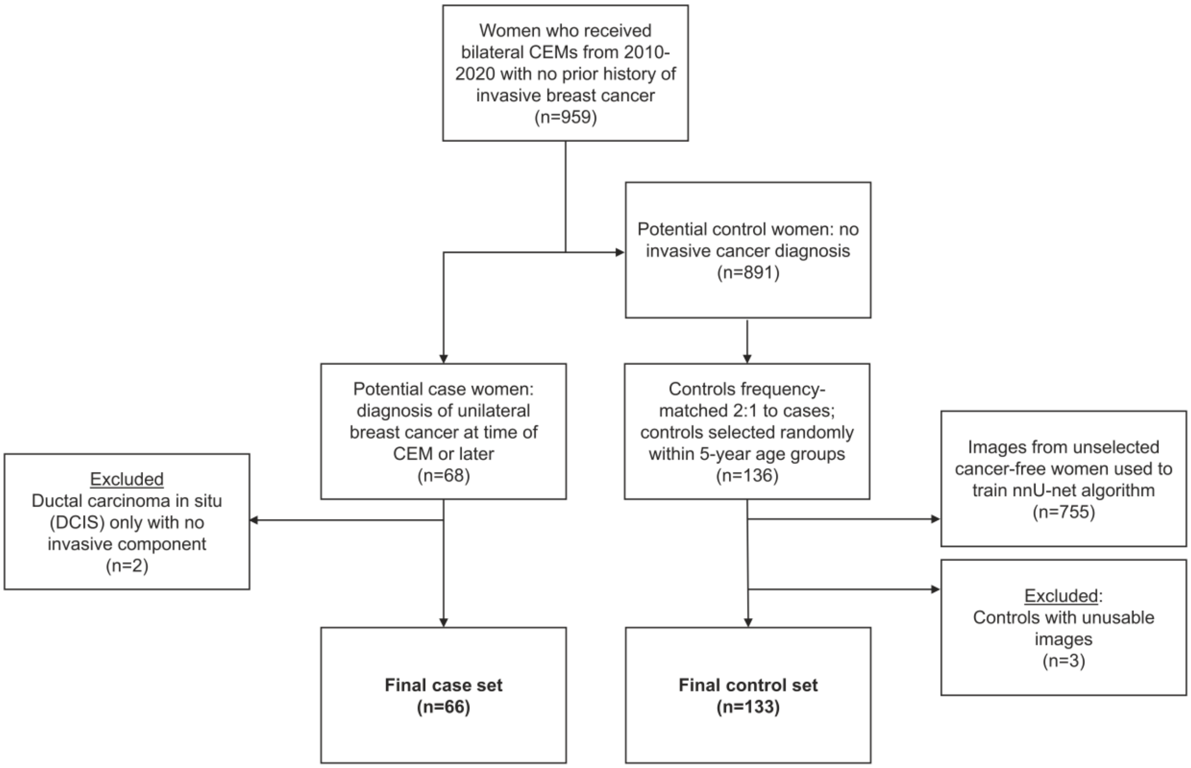

Supplementary Figure 1 Legend. Diagram of case-control sample selection. There were 959 women with CEMs completed at MSK from 2010-2020 with no prior history of breast cancer. From this population, we selected all women with a unilateral invasive cancer diagnosed at the time of or after their CEM date (n = 68). To these 68 case women, we frequency-matched controls at a 2:1 ratio (n=138) based on age at time of mammogram (5-year age groups). The cancer-free women not selected as controls were used for training and tuning the nnU-net algorithm. The algorithm's accuracy was tested on the case-control sample, which was not used to train the measure. Finally, 2 cases were excluded due to a diagnosis of ductal carcinoma *in situ* (DCIS) only with no invasive involvement in accordance with retrospective research protocol, and 3 controls were excluded due to unusable low-energy images.

Supplementary Figure 2

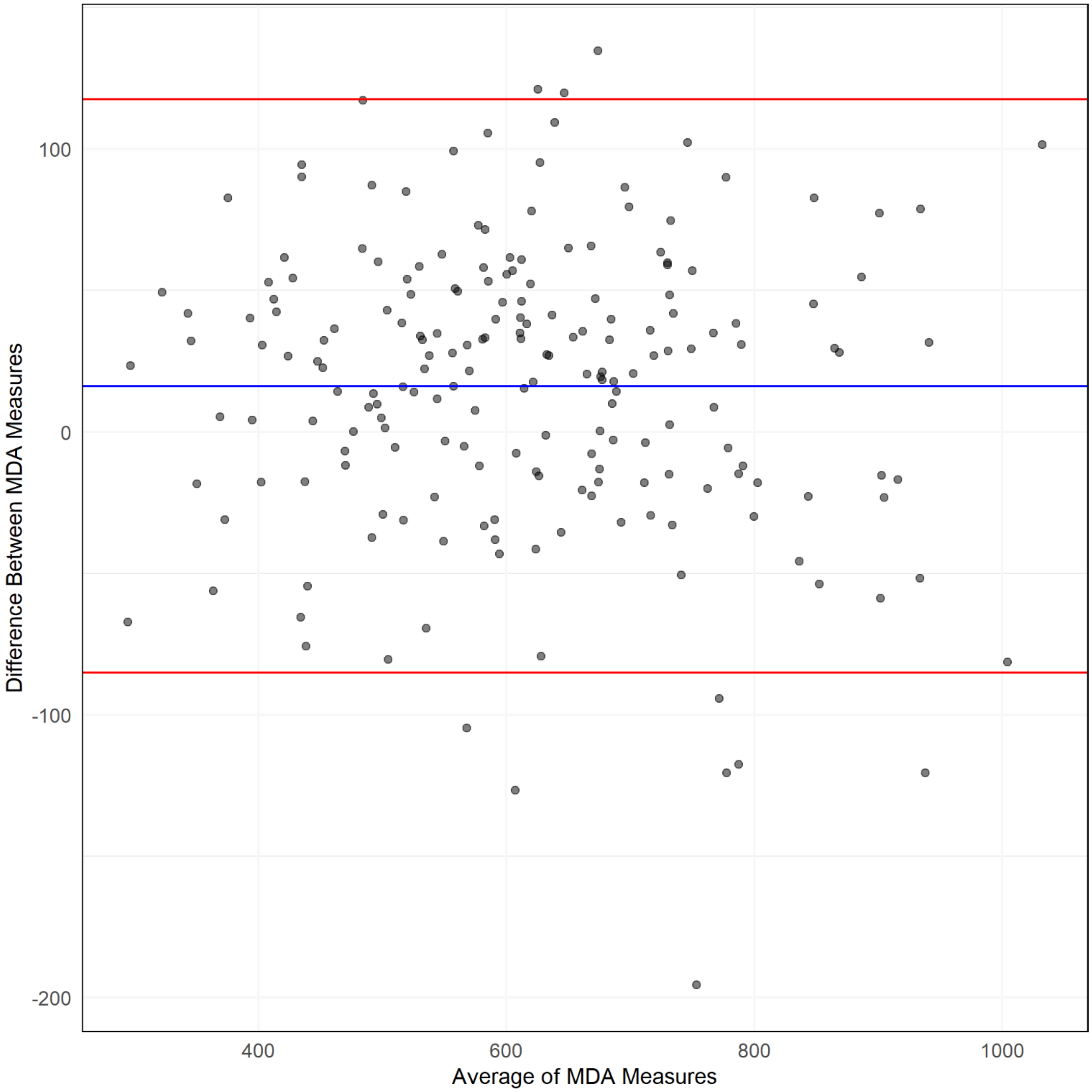

Supplementary Figure 2 Legend. Bland-Altman plot to compare the semi-automated Cumulus mammographic dense area (MDA) measure and the fully-automated nnU-net MDA measure, in pixels. The measures reflect were square-root transformed prior to plotting. Differences were calculated as the nnU-net MDA − Cumulus MDA. The mean difference between the measures (blue line) was 256 pixels. For 11 (5.5%) of the images, the difference between the nnU-net and Cumulus MDA measures were > 1 standard deviation (SD) above or below the mean difference. Trends in differences across average values (x-axis) are not apparent.
